# Psychological and physiological effects of viewing a money plant by older adults

**DOI:** 10.1002/brb3.1359

**Published:** 2019-07-15

**Authors:** Ahmad Hassan, Chen Qibing, Liu Yinggao, Jiang Tao, Guo Li, Mingyan Jiang, Li Nian, Lv Bing‐Yang

**Affiliations:** ^1^ College of Landscape Architecture Sichuan Agricultural University Chengdu China; ^2^ College of Forestry Sichuan Agricultural University Chengdu China; ^3^ College of Forestry Guizhou University Guizhou China

**Keywords:** blood pressure, greenery, high beta, money plant

## Abstract

**Background:**

Observing plants can induce neurophysiological responses that can alleviate stress and reduce anxiety. However, few studies have examined such effects in older adults.

**Methods:**

The physiological and psychological effects of observing nature (visual observation of a money plant) on 50 older Chinese women (age range: 58–90 years, *SD*: 8.5 years) were investigated. The participants observed a healthy money plant in a planter for 5 min; the lack of presence of a plant was used as a control. Physiological measurements were assessed using electroencephalography, and the STAI was used as a psychological assessment.

**Results:**

After a 5‐min observation of a money plant as compared with the control condition, systolic blood pressure significantly decreased, variations in both high alpha and high beta brainwaves were found, and psychological measurements revealed lower anxiety scores.

**Conclusions:**

Our findings indicate that viewing a money plant for 5 min may enhance both psychological and physiological relaxation in older adults.

## 1 BACKGROUND

The aging population is increasing in number worldwide. Almost one in nine people are aged 60 years or above. It is expected that by 2050, the ratio will reach one in every five people (Chan et al., 2017). According to a recent UN report, China is leading in this aging trend, whereby more than 30% of the total population will likely be aged 60 years or greater by 2050 (Banister, Bloom, & Rosenberg, 2012). The elderly population has a relatively high risk of poor mental health, primarily because of social isolation (Cornwell & Waite, 2009). Therefore, in recent years, there has been increasing demand for research into nature‐based therapies that utilize the beneficial effects of the natural environment on well‐being; one example is horticultural therapy. Horticultural therapy is an increasingly popular technique that utilizes various activities, such as viewing, touching, smelling, and tasting, to meet the specific rehabilitative or therapeutic goals of its participants. Among older adults, it is a popular leisure‐time activity that is associated with positive health effects, such as a reduction in anger (Hartig, Evans, Jamner, Davis, & Gärling, 2003). This may be because of the calming effect of nature, as reflected in stress mitigation and recovery of directed attention fatigue (Kaplan, 1995). Horticultural therapy offers health benefits. A recent randomized controlled study found that a significant reduction in pro‐inflammatory cytokine, interleukin‐6 (IL‐6), level was observed in the horticultural therapy group as compared to the wait‐list control group (Ng, Sia, et al., 2018). Pro‐inflammatory cytokines play a key role the pathophysiological of depressive disorder (Liu, Ho, & Mak, 2012) and coronary artery diseases (Ho, Neo, Chua, Cheak, & Mak, 2010). Depression is becoming increasingly prevalent within the elderly population (Vu et al., 2018), and the IL‐6 levels were significantly elevated in elderly with depression (Ng, Tam, et al., 2018). In contrast, antidepressant medication was found to reduce pro‐inflammatory cytokines (Lu et al., 2017) and horticultural therapy demonstrated similar health benefits. A study of the effectiveness of horticultural activities for improving physical health, mental health, and cognitive functioning is, therefore, warranted. If confirmed to be efficacious, this cost‐effective and innovative intervention could represent a nationwide approach to health advancement. It has been noted that healthcare systems insufficiently promote public health (Hancock, 1999). As such, there is a growing need to focus on innovative socio‐ecological techniques to promote health (Maller, Townsend, Pryor, Brown, & St Leger, 2006). Plants, which are readily accessed by community‐dwelling persons, are a perfect source of support in this respect. The focus of this research was to enhance psychological, cognitive, social, and physical functioning and to promote common health and wellness. Electroencephalography (EEG), which records electrical activity inside the brain, is commonly used in research and clinical settings. It is fully non‐invasive and non‐fatiguing, and the recording instrument is reasonably low cost. EEG characterizes electrical fluctuations in the forms of waves, popularly called brainwaves. Studies have assessed the impact of various sensory inputs, such as audition, taste, vision, and olfaction, on the EEG. Previous EEG studies have supported the impact of natural environments on humans. For example, relaxed participants who viewed pictures of a green landscape or urban scenes, or watched outdoor greenery in a seated position, had increased alpha wave activity (Nakamura & Fujii, 1990). Usually, alpha waves are related to physiological arousal (Ulrich, 1981). In contrast, feelings of stress or diminished arousal due to poor concentration during contact with stimuli are associated with low alpha activity. Moreover, EEG is currently being used in experiments on human interfaces (Ajiro, Yamanouchi, Shimomura, Yamamoto, & Kamijo, 2009). EEG is being newly used to investigate certain health disorders, such as stress and anxiety (Angelucci et al., 2014) and it plays a key role in helping stress‐based problems and other brain functions. It is the most accepted and accurate method of assessing stress via brain activity. De Waard (1996) noted that EEG frequencies are usually considered in five different groups: delta waves (4 Hz), alpha waves (8–13 Hz), theta waves (4–8 Hz), beta waves (above 13 Hz), and gamma waves (31–42 Hz; Hassan, Qibing, & Tao, 2018). Each range behaves differently according to various conditions; for example, during a state of workload or stress, alpha waves disappear while beta waves increase in prominence (Angelucci et al., 2014). Furthermore, alpha activity decreases and theta activity increases during high workload or stress (Hankins & Wilson, 1998). In contrast, Brookings, Wilson, and Swain (1996) investigated different types of workload stress using EEG and observed that the EEG is altered during contact with outer environments. Thus, EEG is an appropriate tool by which to expose various brain functions or brain conditions (Hankins & Wilson, 1998). However, there are limitations to the previous research. Most of the previous studies either performed assessments over a short time period (Barnicle & Midden, 2003) or used only simple questionnaires without any evidence‐based support from electrophysiological data. In addition, most previous studies only examined physiological measurements related to visual stimulation with plants in healthy young adults. There have been no investigations of the physiological and psychological effects of visual stimulation with plants in older adults. Further research is, therefore, needed to investigate the effectiveness of “nature therapy” in improving the health of older adults in the Chinese community. Therefore, this study examined the effectiveness of 5 min of plant observation for improving the mental, physical, and cognitive health of community‐dwelling older adults. The focus of this research was to enhance psychological, cognitive, social, and physical functioning and to promote common health and wellness.

## 2 METHODS

### 2.1 Participants

We performed this experiment in a nursing care facility for older individuals in Wenjiang, China. The participants were 50 Chinese women (mean age ± *SD*: 79.2 ± 8.5 years) with hypertension. In this experiment, the participants were chosen according to their availability and on a voluntary basis, after examining their family history and medical records. Participants with good physical health (able to walk by themselves) and good visual perception were selected (i.e., participants with poor vision and poor physical health were excluded). Throughout the experiment, no food or beverages were consumed. All participants provided written informed consent before they took part in the experiment. The study was conducted in accordance with the local ethics committee of the College of Forestry and College of Landscape Architecture, Sichuan Agricultural University, China.

### 2.2 Material

**Figure 1:**
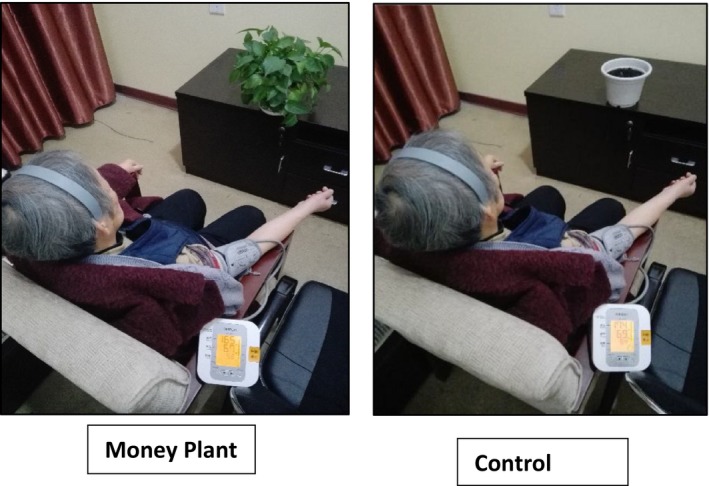
Visual stimulation with money plant (right) and Control (left)

The visual stimulus was a miniature dense money plant, which was approximately 10–15 cm tall and arranged in a plastic pot. An empty pot of similar size and weight was used as a control. The distance from participants' eyes to the plant was adjusted (approximately 38 cm) according to the given plant and the height of participant, as shown in Figure 1. The average light intensity, temperature, and humidity were maintained at 23°C, 40%, and 500 lux, respectively.

### 2.3 Protocol

Participants were randomly divided into a plant group (25 participants) and a control group (25 participants). On the first day, participants in the first group observed a money plant for 5 min and the other group received visual stimulation without any plant for 5 min. On the next day, plant and control groups switched their activities.

### 2.4 Procedure

We recorded blood pressure before and after visual stimulation, whereas EEG was recorded throughout the 5‐min visual observation. Participants completed STAI questionnaires both before and after receiving visual stimulation. The STAI was used to evaluate participants' anxiety levels (Spielberger, Gorsuch, Lushene, Vagg, & Jacobs, 1983). The STAI comprises 20 questions (e.g., addressing anxious feelings); participants are asked to make ratings on four scales. Participants' STAI scores were calculated by summing their ratings of the 20 questions. Participants with higher scores exhibited higher state anxiety. Physiological measurements consisted of EEG (NeuroSky MindWave EEG headset; Beijing Oriental Creation Technology Co.) and blood pressure (Sphygmomanometer: Omron HEM‐7011; Omron). The EEG sensor was placed on the participant's head at the FP1 position to record high alpha and high beta waves. The EEG headset consisted of an EEG electrode (sensor), an ear‐clip, Bluetooth (USB) interface, and a headband. EEG raw data were sampled at a rate of 512 Hz; other values were sampled every second. The EEG data were evaluated within 1‐min intervals and 5‐min averages of high alpha and high beta waves were used when comparing responses to the two conditions.

### 2.5 Statistical analysis

SPSS 16.0 software (SPSS Inc.) was used for statistical analyses. Repeated‐measures analysis of variance (ANOVA) and paired *t* tests were used to compare mean differences between physiological data and to assess carry‐over effects, while the Wilcoxon signed‐rank test was used to compare psychological data. For all tests, a significance level of *p* < .01 was used.

## 3 RESULTS

**Figure 2:**
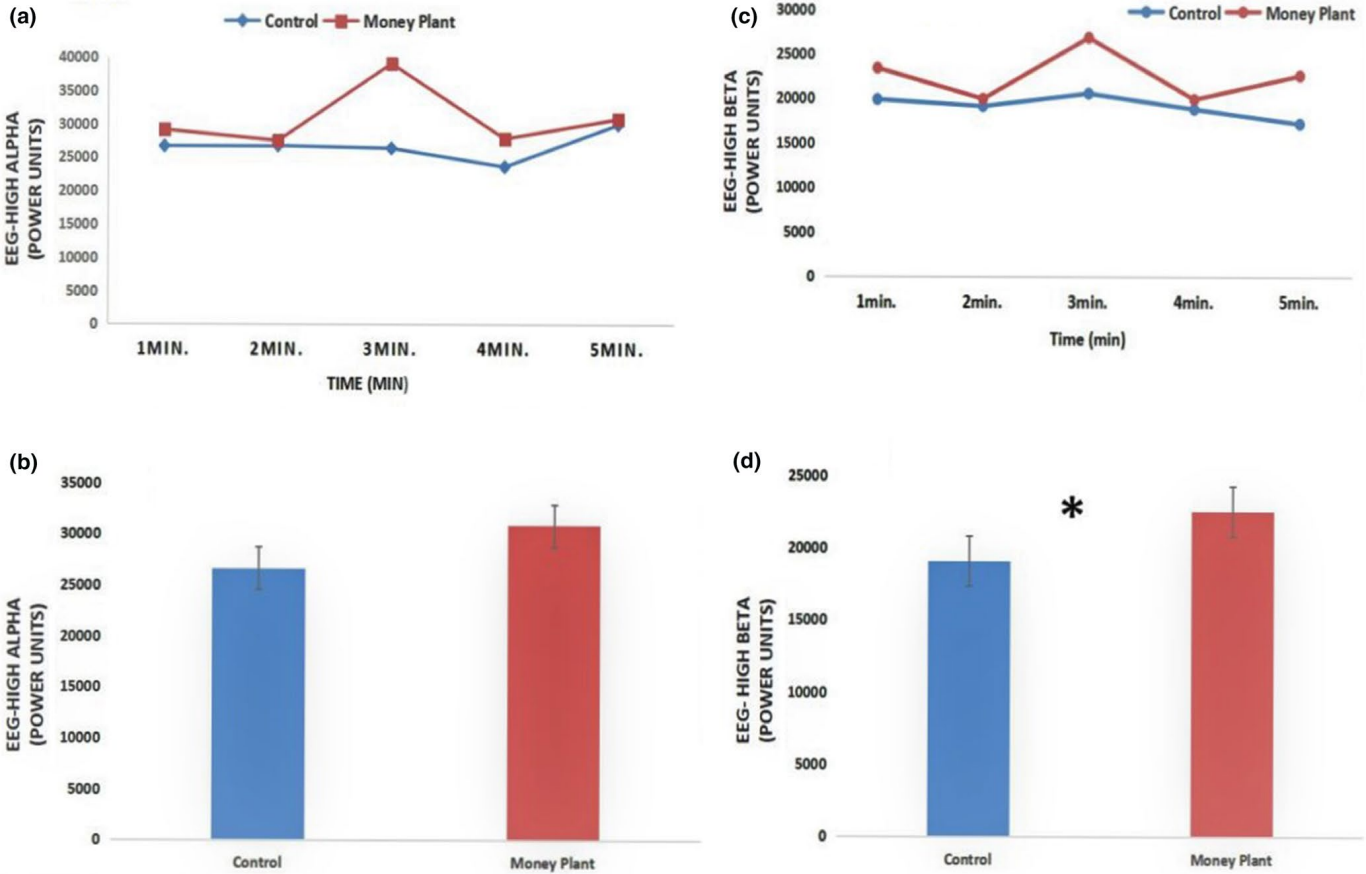
Comparison of mean values of high alpha and high beta brainwaves (power units) between money plant and control. (a) 1–5 min mean values of high alpha brainwave (power units); (b) overall mean value of high alpha brainwave (power units); (c) 1–5 min mean values of high beta brainwave (power units); (d) overall mean value of high beta brainwave (power units). *N* = 50, mean ± *SD*, paired *t* test, * *p* < .01

Systolic blood pressure was significantly (*p* < .01) lower after a 5‐min visual stimulation with a money plant as compared with the control (Table 1). However, diastolic blood pressure did not differ significantly between conditions. The total state‐trait anxiety scores (STAI) were significantly lower after 5 min of viewing a money plant than after the control (money plant: 34 ± 4.33; control: 40 ± 7.75; *p* < .01). Differences were non‐significant before each task (Table 1). EEG high alpha and high beta waves were assessed every minute as reflecting neurophysiological activity. Most of the high alpha and high beta mean values increased during visual stimulation with a money plant as compared with the control (Figure 2). Further, paired *t* tests showed that the overall mean high beta amplitude significantly increased. However, repeated‐measures ANOVA revealed no significant differences in overall mean high alpha amplitude between conditions (*F *(4) = .69, *p* = .59). Similarly, high beta amplitude did not differ over time intervals between conditions (*F *(4) = .49, *p* = .73).

**Table 1:** Mean ± *SD* of participants before and after performing tasks

Measurements	Money plant (Mean ± *SD*)	Control (Mean ± *SD*)	*p*‐value
Systolic blood pressure (mmHg)	164.53 ± 6.16	172.13 ± 6.46	.01
Diastolic blood pressure (mmHg)	67.3 ± 9.05	68.2 ± 5.77	.67
STAI score			
Before	38.4 ± 5.7	39.5 ± 7.1	.55
After	34.0 ± 4.3	40.0 ± 7.7	.01

## 4 DISCUSSION

We investigated psychological and physiological effects of viewing a money plant for 5 min in comparison with viewing no plant as a control. Compared with the control condition, observing a money plant significantly decreased systolic blood pressure. Systolic blood pressure reduction indicates a state of calmness and lack of trauma in older adults. Our findings are broadly consistent with other experiments that examined physiological responses of older adults to gardening activity (Hassan et al., 2018). Various clinical and laboratory studies have found that visual stimulation via plants for <5 min has a positive impact on health, such as by reducing blood pressure, heart rate, and muscle tension (Ulrich, 1981, 1991). However, there are many differences between our study and these prior studies; for example, our study included older women, but only young adults were included in previous experiments. Additionally, the reduction in blood pressure over the time period of our study also differed from previous studies. EEG is used in many research and clinical settings to illustrate differences in neural activity. Percepts of audition, vision, olfaction, and taste alter brainwave activity (Ajiro et al., 2009). Recently, EEG studies have been conducted regarding the relief of stress and anxiety (Angelucci et al., 2014). In particular, different waves in the human brain are associated with different mental states; for example, alpha waves occur during relaxation. In our study, high alpha activity was greater during visual stimulation with a money plant as compared with the control. Thus, calmness improved after viewing the money plant versus after viewing the control stimulus. Our findings are partly consistent with a previous study, in which visual stimulation with natural environments significantly altered EEG activity and lowered sympathetic nervous system activity. Results of this previous study indicated that visual stimulation with a natural hedge in comparison with viewing a photo of a similar concrete wall produced greater alpha and beta activity (Nakamura & Fujii, 1992). Studies have also revealed that feelings of joy and sadness are also related to increased or decreased alpha wave activity, respectively (Kostyunina & Kulikov, 1996). Moreover, higher alpha wave activity is correlated with various emotional states, such as tremor, agitation, aggression, loss of appetite, and anger. In addition, during quiet conditions, alpha activity dominates; such activity denotes calmness or relaxation (Başar, 2012). In contrast, lower levels of alpha activity are usually associated with transient ischemic attacks (Madkour et al., 1993). Greenery promoted alpha activity in older adults. Similarly, high beta activity increased during visual stimulation with a money plant as compared with the control. The significant increase in beta activity indicated that participants were alert and in a highly active state. Usually, increased beta activity occurs in highly active states, whereas decreased beta activity occurs during a state of drowsiness (Lee, Lee, & Chung, 2014). Our results are partly consistent with a previous study of gardening activity involving potted plants, which showed high beta activity while transplanting plants versus filling pots (Yamane, Kawashima, Fujishige, & Yoshida, 2002). Moreover, playing sports, engaging in intense conversation, attending a job interview, or giving a speech all enhance beta wave activity. Furthermore, beta activity also occurs when a person is externally focused and relaxed (Jemmer, 2009). Therefore, we conclude that viewing a money plant affected beta wave activity. Regarding the psychological evaluation, STAI anxiety scores were lower after visual stimulation with a money plant. Therefore, the psychological benefits of greenery for older adults are significant (i.e., decreased anxiety), and an indoor green environment can enhance spiritual health (Qin, Sun, Zhou, Leng, & Lian, 2014). The supportive effects of viewing a money plant indicate that it is an easy, convenient, and effective method by which to enhance the well‐being of older adults living in nursing homes. The current study has some limitations; for example, a relatively small sample of only 50 older adults was used. Future studies should use larger samples and better control groups. In addition, studies of different indoor plants are warranted. Functional near‐infrared spectroscopy (fNIRS) is a non‐invasive neuroimaging technology that maps the functions of the cerebral cortex by measuring hemodynamics and demonstrates cost‐effectiveness (Lai, Ho, Lim, & Ho, 2017). A recent fNIRS study showed that urban scene led to significant increase of oxyhemoglobin on the right area of the prefrontal cortex as compared to the garden scene (Yu, Ang, Ho, Sia, & Ho, 2017) and further research is required to assess the effect of plant viewing by fNRIS. However, we do not have a full understanding of the various EEG results. These findings potentially resulted from the differences in the extent to which the participants liked or disliked the natural environment. Plants play an important role in improving the health of older adults in stressful environments; thus, landscape designers should incorporate indoor plants (green spaces) inside the living spaces of elderly care facilities, to enhance the quality of life of older adults.

## 5 CONCLUSION

We conclude that 5 min of visual stimulation with a money plant induces psychophysiological relaxation in older adults.

## CONFLICT OF INTEREST

Authors declare that they have no competing interests.

## AUTHORS' CONTRIBUTIONS

Ahmad Hassan contributed to data acquisition, statistical analysis, interpretation of the results, and manuscript preparation. Jiang Tao, Guo Li, Mingyan Jiang, Li Nian, Lv Bing‐Yang help out in final revision of manuscript.

## DATA AVAILABILITY

Data is confidential.

## ETHICS APPROVAL AND CONSENT TO PARTICIPATE

Subject's written informed consent was obtained. This study arranged with the approval of local Ethics Committee College of Landscape Architecture, Sichuan Agricultural University, China.

## CONSENT FOR PUBLICATION

Subject's written informed consent was obtained for the publication of images.
